# Clopidogrel‐induced liver damage: A case report and review of the literature

**DOI:** 10.1002/ccr3.3324

**Published:** 2020-09-16

**Authors:** Vahid Eslami, Azin Gheymati

**Affiliations:** ^1^ Shahid Beheshti University of Medical Sciences, Cardiovascular Research Center Tehran Iran; ^2^ Tehran University of Medical Sciences, School of Pharmacy Tehran Iran

**Keywords:** cholestasis, hepatocellular, icterus, clopidogrel

## Abstract

Liver damage is a rare side effect of clopidogrel. That is reversible in most cases. Considering the widespread use of this medication in cardiovascular diseases, the management of hepatotoxicity requires further meticulous investigation.

## Graphical abstract

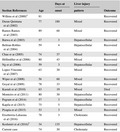

## 1 BACKGROUND

The liver has a major role in the elimination and excretion of many drugs, which can sometimes lead to chemical damage. Epidemiologic studies have revealed that 1 in every 1000 individuals suffers from drug‐induced liver injury (DILI); this damage can vary from mild laboratory abnormalities to acute liver failure.^1^, ^2^ Clopidogrel is an antiplatelet medication from the thienopyridine group which is commonly used to prevent clot formation in coronary, cerebral, and peripheral vascular diseases and has led to decreased mortality and reinfarction in patients with acute coronary syndrome. With increasing use of clopidogrel in recent years, the reports of clopidogrel‐induced hepatotoxicity are also increasing.. Except two reported cases of cholestasis alone, liver injury due to clopidogrel has been in the form of a hepatocellular or mixed cholestatic/hepatocellular pattern.^3^, ^4^, ^5^ In this case report, an individual with severe liver injury due to clopidogrel is assessed and there is also a literature review.

## 2 CASE PRESENTATION

The patient was a 78‐year‐old man who had successfully been treated by thrombolysis (Alteplase, Boehringer‐Ingelheim) for anterior STEMI, before undergoing angiography and angioplasty on the LAD with a drug‐eluting stent the next day. His only risk factor for coronary disease was a previous history of smoking. Echocardiography demonstrated, severe LV systolic dysfunction, (EF = 25%‐30%), with Large LV Clot (16 × 7 mm^2^) in apex and no significant valvular disease. The patient was discharged in a stable condition with normal laboratory findings and prescribed the following medications:

Tab ASA 80 mg/daily, tab Clopidogrel 75 mg/daily (sanofi), tab carvedilol 6.25 BD, tab captopril 25 mg BD, tab pantoprazole 40 mg/daily, tab warfarin 5 mg/daily.

At the time of discharge, the following laboratory data were noted as follows:

Wbc = 10 200 (4.5 −10.8 × 10^9^/L), Hgb = 14.5 g/dL, plt = 290 000, AST = 135 μ/L (10‐42), ALT = 37 μ/L (7‐45), bilirubin µmol/L (total = 1.2, direct = 0.3), cr = 1.05mg/dL.

40 days after angioplasty, the patient returned complaining of icterus, itching, and decreased appetite. The patient had no recent history of fever, abdominal pain, weight loss, nausea, vomiting, urine or stool discoloration, or bleeding from any site in the body. He had no history of using over the counter drugs, such as analgesics or a anxiolytics, herbal remedies, or alcoholic beverages. In the physical examination, the vital signs were stable and patient did not appear ill or toxic and the general condition was good and has no complaint except icterus and loss of appetite. None of the stigmata of chronic liver disease were observed; the abdomen was soft, and there was no sign of ascites or organomegaly. Cardiac and neurological examinations did not reveal any abnormalities.

A gastrointestinal consultation was requested for the patient, and further tests were conducted as shown in Table 1

**Table 1:** Patient's laboratory data

Bilirubin (µmol/lit)	Total (6.1), direct 5.7)*
ALT (U/L)	266*(7‐45)
AST (U/L)	122* (10‐42)
GAMA GT (U/L)	930(11‐50)*
ALK PHOSP ( IU/L)	545(40‐130)*
ALBUMIN (g/DL)	4 g/dL (3.5‐5.5)
IgG(SERUM) mg/dL	1230(700‐1600)
INR	2.7
IgG 4 SUBCLASS (mg/dL)	80(4‐86)
AMA‐M2	NEGATIVE titer less than 1:40
SERUM TOTAL IgG (mg/dL)	12.61 (7‐16)
IgM (mg/dL)	0.87 (0.4‐2.3)
FANA	Negative
Serum protein electrophoresis Hepatitis A HBS Ag HCV Ab Hepatitis E CEA CA19‐9	Normal Negative Negative Negative Negative NL NL

Immunologic and serological tests for hepatitis A, B, C, and E and tumor markers were negative (Table 1); abdominopelvic ultrasound showed no lesions, and subsequent MRCP displayed normal size and shape of the intrahepatic and extrahepatic biliary tracts as well as normal wall thickness and appearance of the gall bladder, without any lesions or filling defects. The pancreatic ducts were normal.

Based on the lack of anatomical lesions, a diagnosis of liver injury with a hepatocellular, cholestatic, or mixed pattern due to autoimmunity or, more probably, drug‐induced, was considered. Bearing in mind the patient's most recent echocardiography, which had shown relatively improved cardiac function without the presence of clots in the left ventricle (EF = 35%), medications were discontinued based on their likelihood to cause liver damage, so statins were discontinued initially, followed by captopril and, lastly, warfarin. Despite these measures, no improvement in the laboratory findings or the patient's jaundice was observed and there was actually an increase in the bilirubin level.

Bilirubin (total = 13, direct =8.8), AST = 130, ALT = 250.

After [witnessing] the rise in bilirubin, clopidogrel was substituted by ticagrelor; subsequently, over the next two weeks, the patient's jaundice improved and the laboratory results returned to normal.

Bilirubin (total = 1.05, direct = 0.7), AST = 41, ALT = 52, alkaline phosphatase = 141.

Medications other than clopidogrel were restarted gradually with 1 week of intervals in between and over a follow‐up period of two months, normalization of the laboratory data and his general condition was observed.

## 3 DISCUSSION

Clopidogrel is highly effective in the treatment of cardiovascular disease, but occasional side effects, such as rash, gastrointestinal problems, and bleeding, have been reported after its administration. In recent years, some researchers have cited hepatotoxic side effects related to this drug;^6^, ^7^ more specifically, 20 such cases have been reported in the literature (Table 2). Out of the 20 cases so far reported, 8 were females and 12 were males with a mean age of 67.8 years (range 34‐89) at the time of the diagnosis. On average, the hepatotoxicity developed 35 days after starting the clopidogrel (range 3‐180 days). In the literature, the most common pattern of liver damage was mixed (11 cases), while 5 cases showed solely hepatocellular involvement and two displays only cholestatic injury.^4^, ^8^, ^9^ Most studies have indicated the survival of the majority of patients and improvement of laboratory results within 1 week to 6 months of discontinuing the medication. In our presented case, improvement of laboratory findings took 2 weeks. To our knowledge, there has only been a single death reported due to the hepatotoxic side effects of clopidogrel.^10^

**Table 2:** Side effects related to clopidogrel based on the literature^5^

Section References	Age	Days at	Liver injury	Outcome
		onset	pattern	
Willens et al (2000)^3^	81			Recovered
Duran Quintana et al (2002)	77	180	Mixed	Recovered
Ramos Ramos et al (2003)	89	60	Mixed	Recovered
Batwa et al (2003)	57	3	Hepatocellular	Recovered
Beltran‐Robles et al (2004)	59	4	Hepatocellular	Recovered
Chau et at (2005)	74	37	Mixed	Recovered
Höllmüller et al (2006)	80	43	Mixed	Recovered
Ng et al (2006)	59	3	Hepatocellular	Recovered
Lopez‐Vincente et al (2007)	63	30	Mixed	Recovered
Wiper et al (2008)^5^	56	60	Mixed	Recovered
Goyal et al (2009)^5^	78	33	Mixed	Recovered
Kastali et al (2010)^5^	63	19	Mixed	Died
Monteiro et al (2011)^5^	80	30	Hepatocellular	Recovered
Pegram et al (2014)^5^	57	3	Hepatocellular	Recovered
Kapila et al (2015)^5^	75	5	Hepatocellular	Recovered
Pisapia et al (2015)^5^	53	3	Mixed	Recovered
Etxeberria‐Lekuona et al (2016)^5^	78	3	Cholestatic	Recovered
Keshmiri et al (2016)^4^	34	135	Hepatocellular	Recovered
Current case	74	30	Cholestatic	Recovered

The exact cause of liver injury due to clopidogrel remains undetermined, but two possible mechanisms consisting of a direct dose‐dependent reaction or an idiosyncratic non–dose‐dependent reaction have been suggested.^11^

In our case, hepatotoxicity was observed clinically nearly 1 month after the initiation of clopidogrel because the patient did not have any complaint of icterus or pruritus before one month, whether he has not been visited or performed laboratory evaluation before his first visit; he demonstrated no signs of hypersensitivity, such as rash, arthralgia, or eosinophilia, and the clinical presentation pointed (more) toward a dose‐dependent reaction.

Clopidogrel is a prodrug converted to its active form, which consists of MERCAPTO group, by various cytochromes in the liver, including CYP 4A3 and CYT P 450.^11^, ^12^ These active metabolites are responsible for the antiplatelet effects as well as the hepatotoxic effects of the medication due to inhibition of cell glutathione. Medications that stimulate CYP 4A3, such as rifampin, can increase the harmful effects of clopidogrel on the liver; moreover, one significant finding on biopsies carried out by various investigators was that the greatest damage was observed in zone 3 of the liver, which indicates the potential involvement of CYT P 450 in the pathogenesis of liver injury. The inhibition of the cytochrome P 450 isoenzyme by clopidogrel is dependent on time, dosage, and NADPH. Drugs such as captopril, hydrochlorothiazide, and allopurinol can lead to cholestasis via this pathway. By affecting some isoenzymes in the cytochrome P 450 and CYP 4A3 pathways, interactions between clopidogrel and omeprazole or certain statins, such as atorvastatin, may lead to liver damage.^13^

In summary, due to the widespread use of clopidogrel in various diseases, it is prudent to bear in mind the less common side effects of this medication, particularly when there is a possibility of drug interaction.

## CONFLICT OF INTEREST

None declared.

## AUTHOR CONTRIBUTIONS

Dr Azin Gheimati: has been as the pharmacotherapy consultant during the patient management and has reviewed the article, and doctor Vahid Eslami: has been the responsible physician of the case and has written the article.

## COMPLIANCE WITH ETHICAL STANDARDS

Patient management and data gathering were based on the ethical standards of Shahid Beheshti Medical University. Written informed consent was obtained from the patient. The consent form is held by the authors.
